# Eleven years’ experience with Intrathecal Baclofen – Complications, risk factors

**DOI:** 10.1002/brb3.965

**Published:** 2018-03-30

**Authors:** Elke Pucks‐Faes, Gabriel Hitzenberger, Heinrich Matzak, Elena Fava, Giulio Verrienti, Ilse Laimer, Josef Fritz, Leopold Saltuari

**Affiliations:** ^1^ Department of Neurology Hochzirl Hospital Zirl Austria; ^2^ Department of Neurosurgery Medical University Innsbruck Innsbruck Austria; ^3^ Department of Medical Statistics, Informatics and Health Economics Medical University Innsbruck Innsbruck Austria; ^4^ Research Unit for Neurorehabilitation South Tyrol Italy

**Keywords:** autonomic nervous system diseases, baclofen, brain injuries, sympathetic nervous system, traumatic

## Abstract

**Objective:**

Treatment with intrathecal baclofen (ITB) is commonly used in patients with severe spasticity. However, complications may occur after implantation of the ITB‐device, albeit mainly procedure‐ and device‐related problems. The aim of the study was to assess surgical‐ as well as catheter‐ and pump‐related complications and define their risk factors.

**Methods:**

We retrospectively evaluated all patients with an implanted ITB‐device who were treated at the Department of Neurology, Hochzirl Hospital, Zirl, Austria, between 2006 and 2016.

**Results:**

Twenty‐nine of 116 (25%) patients experienced 32 complications: 5 procedure‐ and 27 device‐related (4 pump‐ and 23 catheter‐associated) problems occurred. Risk factors for sustaining any complication were a spinal localization of lesion (odds ratio [OR] OR 2.71, *p* = .021), other catheter types than an Ascenda^®^ catheter (OR 3.87, *p* = .041), a lower modified Rankin Scale (median 4 vs. 5; OR 2.86, *p* = .015) and a higher Barthel Index (median 53 vs. 0; OR 2.84, *p* = .006). The median time from the last ITB‐related surgery to the first complication was 18 (IQR 1‐57) months. Overall, 47% complications occurred within the first year after any surgical procedure regarding the ITB‐device, thereof 25% within the first month.

**Conclusions:**

Procedure‐ and device‐related complications are frequent after implantation of an ITB‐device with catheter‐associated complications as the most frequently encountered problems. Patients with a spinal origin of spasticity, a lower modified Rankin Scale and a higher Barthel Index have a higher risk to sustain a complication.

## 1 INTRODUCTION

Intrathecal baclofen (ITB) has successfully been used in patients with severe spasticity of different etiologies for more than 30 years (Albright, Barron, Fasick, Polinko, & Janosky, 1993; Albright, Cervi, & Singletary, 1991; Armstrong et al., 1997; Becker, Alberti, & Bauer, 1997; Becker, Sure, Petermeyer, & Bertalanffy, 1999; Coffey et al., 1993; Dario, Di Stefano, Grossi, Casagrande, & Bono, 2002; Meythaler, DeVivo, & Hadley, 1996; Meythaler, Guin‐Renfroe, Grabb, & Hadley, 1999; Meythaler, McCary, & Hadley, 1997; Penn & Kroin, 1985; Penn et al., 1989; Rawicki, 1999; Rifici et al., 1994). Treatment of severe spinal as well as supraspinal spasticity with oral antispastic medication often results in an insufficient response and is limited by intolerable side effects. The therapeutic utility of ITB in the management of severe spasticity has been repeatedly demonstrated, but nevertheless, complications may occur after the ITB‐pump has been implanted and therefore decrease therapeutic effects. The classification often used in the literature comprises the differentiation between procedure‐related, device‐related and drug‐related complications (Borrini et al., 2014; Turner, Sears, & Loeser, 2007). A review made in 2010 only focused on procedure‐ and device‐related complications of ITB‐administration (Stetkarova, Yablon, Kofler, & Stokic, 2010): 558 complications were reported after 1,362 pump implantations, of which 27% were related to surgical procedures, 7% to pump problems, and 66% to catheter malfunctions.

In a prospective, observational cohort study in 158 adults treated with ITB therapy and followed up for 1 year, 18% experienced 38 adverse events (Borrini et al., 2014). Motta and coworkers analyzed complications and risk factors in 200 consecutive children and adolescents after intrathecal baclofen pump implantation (Motta, Buonaguro, & Stignani, 2007). A statistically significant correlation between the occurrence of a complication and patients with an Ashworth Scale higher than 3 and an age of 10 years or younger was found. In the largest series in 430 consecutive children treated with ITB in a 14‐year period, 137 complications occurred in 25% (Motta & Antonello, 2014).

The purpose of this study is to give a detailed account of the most frequently encountered complications, catheter‐ and pump‐associated problems and define their risk factors.

## 2 MATERIALS AND METHODS

We retrospectively evaluated all patients with an intrathecal ITB‐device, who were treated at our Department of Neurology, between 01.01.2006 and 31.12.2016. Patients of any age and receiving care bothin inpatient as well as in outpatient hospital setting were included.

ITB‐pump‐related surgical interventions were classified as “primary implantation procedure” (pump implantation for the first time) and “replacement procedure” (pump surgery: replacement due to end of battery life 72 months after implantation at the earliest or revision because of a complication; catheter‐only‐revision after a complication; exchange of the entire system) according to earlier published studies (Awaad et al., 2012; Borrini et al., 2014). The retrospective analysis was done by reviewing patient's medical history and the data provided in our ITB‐dedicated register, which was implemented in 01.01.2006. Exclusion criteria were insufficient patient data. The majority of patients were admitted for ITB‐evaluation, presurgical and postsurgical care as well as follow‐up after implantation of the ITB‐pump. The remaining patients were referred because of an occurring complication for diagnostic work‐up and treatment but followed up at their local district hospital. According to earlier published studies, complications were divided into procedure‐related and device‐related complications, the latter into catheter‐ and pump‐associated complications (Guillaume, Van Havenbergh, Vloeberghs, Vidal, & Roeste, 2005; Kolaski & Logan, 2007; Rawlins, 2004). Procedure‐associated problems were defined as related to the surgical intervention occurring within the first 2 months after a surgical intervention and thus most likely being associated with the surgical intervention, such as scar complications, subcutaneous seroma, infections, or cerebrospinal fluid (CSF) leakages. Device‐associated problems include complications related to the catheter or the pump and were classified as “early” when they occurred within the first 2 months or “late” when occurring later than 2 months after the surgical intervention.

We collected clinical data in all included patients: demographic information, spinal versus supraspinal localization of lesion responsible for the spasticity and data of any previous ITB‐device‐related surgery. Moreover, the type of the implanted catheter (Ascenda^®^ catheter/Model 8781 vs. other catheter types) and the following parameters at steady‐state of ITB were assessed: Ashworth Scale, modified Rankin Scale, Barthel Index, the median ITB‐dosage, median ITB‐concentration, flow rate of ITB, mode of application, flow rate of ITB, and last follow‐up. In patients admitted more than once, the follow‐up was defined as period in months between the first and last consultation at our department. In patients with complications, we analyzed the number and type of complications, as well as the type of intervention. Patients with a suspected complication underwent a comprehensive diagnostic work‐up including the evaluation of the clinical response to ITB, laboratory test and imaging (depending on the clinical situation conventional x‐ray, computer tomography without or with contrasting agent, magnet resonance imaging or fluoroscopy). If the aetiology of the complication still remained unclear, a surgical exploration was done. In patients with a complication, we calculated the duration of the hospital stay from admission to the day of a consecutive intervention (revision surgery, surgical exploration, or nonsurgical intervention). Moreover, the time from the initial ITB‐device implantation and from the last ITB‐related surgery respectively to any occurring complication during the observational period was calculated in all patients with complications. In addition, risk factors for sustaining a complication were analyzed.

### 2.1 Statistical analysis

Data were summarized in cross tables, and medians and interquartile ranges were calculated for ordinal variables, such as modified Rankin Scale, Barthel Index, or Asworth Scale. Complication and incident rates for the occurrence of complications were calculated and odds ratios comparing different subgroups regarding complication frequency were given. Since there were three patients with two complications, statistical analysis was conducted with cases including the second complication. Odds ratios for ordinal risk factors were obtained by dichotomizing the respective risk factor using the overall median value as the cut‐off threshold. The influence of risk factors on the occurrence of complications was tested by chi‐squared test for nominal and by Mann–Whitney U test for ordinal risk factors. A significance level of α = .05 (2‐tailed) was applied. Statistical analyses were performed using SPSS, version 22.0 (IBM Corp., Armonk, NY, USA).

According to our country's law on retrospective research, this study did not require the approval of the ethics committee, but was performed based on ethical standards of the “World Medical Association Helsinki Declaration” (https://www.wma.net/policies-post/wma-declaration-of-helsinki-ethical-principles-for-medical-research-involving-human-subjects/).

## 3 RESULTS

One hundred and thirty‐six patients with an ITB‐pump were treated within the given 11‐year period at our neurorehabilitation department. One hundred and sixteen patients (77 men and 39 women, mean age at implantation 39 ± 16.7 years, range 2‐77 years) entered the final analysis. The location of lesion responsible for the spasticity was supraspinal in 73/116 (60%) patients with traumatic etiology in 34/116, hypoxic in 16/116, and 23/116 had other etiologies (seven infantile cerebral palsy, six intracerebral hemorrhage, four ischemic lesions, three subarachnoidal hemorrhage, one stiff person syndrome, one extra‐/pontine myelinolysis, one undetermined neurodegenerative disease). Forty‐three of 116 (40%) patients had a spinal lesion as origin of spasticity (18 traumatic, 14 multiple sclerosis, four sporadic spastic paralysis, three hereditary spastic paralysis, one disc herniation, one ischemic lesion, one myelopathy due to extradural granuloma, one undetermined spastic paraplegia). Patients’ characteristics and further clinical information are displayed in Table 1.

**Table 1:** Demographic patients’ characteristics and further clinical information

Patient characteristics	
Age at implantation (mean ± standard deviation; years)	39 ± 16.7
Sex (women/men; *n*)	39/77
Pathologic disorders	
Supraspinal [*n*/%]	
Traumatic brain injury	34/29%
Cerebral hypoxia	16/14%
Cerebral palsy	7/6%
Intracerebral hemorrhage	6/5%
Ischemic stroke	4/3%
Subarachnoidal hemorrhage	3/3%
Stiff person syndrome	1/1%
Extra‐/pontine myelinolysis	1/1%
Unknown neurodegenerative disease	1/1%
Spinal [*n*/%]	
Traumatic spinal injury	18/15%
Multiple sclerosis	14/12%
Spastic paralysis	7/6%
Disc herniatio	1/1%
Ischemic lesion	1/1%
Myelopathy due to extradural granuloma	1/1%
Spastic paresis of unclear etiology	1/1%
Investigated parameters^a^	
Median modified Rankin Scale (IQR)	5 (4‐5)
Median Barthel Index (IQR)	0 (0‐54)
Median ITB‐dosage (IQR) [μg/day]	200 (129‐320)
Median ITB‐concentration (IQR) [μg/ml]	2,000 (500‐2,000)
Median flow rate (IQR) [μl/hr]	6.3 (3.5‐10.4)
Median Ashworth Scale (IQR)	4 (3‐4)
Catheter type [Ascenda^®^, Model 8,781/other types; *n*/%]	39/77

IQR, interquartile range; ITB, intrathecal baclofen.

a The medians of the investigated parameter were calculated on basis of the patient population.

One hundred and eight of 116 primary implantation procedures as well as surgical management of all complications were performed at the Department of Neurosurgery, 8/116 ITB‐pumps were implanted in another neurosurgical department. Overall, 143 surgical procedures were performed period within the 11‐year observational period: 82 implantations of the ITB‐pump for the first time (the remaining 34 implantations were done before 01.01.2006), 35 replacement‐surgeries due to end of battery life (one time in 29 patients, two times in three patients) and 26 revision procedures due to a complication (two times in three patients each). Seventy‐seven intrathecal catheter types other than an Ascenda^®^ (Model 8781) catheter were implanted. The median Ashworth Scale of all included patients at steady‐state of ITB was 4 (interquartile range [IQR] 3‐4), the median modified Ranking Scale 5 (IQR 4‐5) and the median Barthel Index 0 (IQR 0‐54). The median ITB‐dosage was 200 μg/d (IQR 129‐320) at steady‐state of ITB, the median ITB‐concentration 2000 μg/ml (IQR 500‐2000; 82/116 2000 μg/ml, 30/116 500 μg/ml, 2/116 1000 μg/ml, 1/116 1250 μg/ml, 1/116 250 μg/ml) and the median flow rate 6.3 μl/hr (IQR 3.5‐10.4) (see also Table 1). The mode of application after reaching steady‐state of ITB was continuous in 95% and flex mode in 5% patients. The median follow‐up was 42 months (IQR 17‐91, mean 56 months), comprising 506 pump years. Seven patients died during follow‐up (one suicide, three severe pneumonia, three sepsis). There was no permanent morbidity or death related to the ITB‐pump‐complication.

### 3.1 Complications

Thirty‐two complications occurred in 29/116 patients during the 11‐year study period. Three patients experienced two complications each. Five of 32 (16%) complications were related to surgical procedures and 27/32 (84%) were device‐related (23 catheter‐associated problems, four pump‐associated problems). Procedure‐related complications contained two pump site infections and three CSF leakages. Catheter‐related problems were: nine dislocations (in eight patients), three CSF leakages, five disconnections, three kinks, one break, and two unknown catheter dysfunctions. Pump‐related problems were caused by hypermobility (1/32) and by pump site infections (3/32 in three patients). Six of 27 device‐associated problems were early complications (three CSF leakages, three pump site infections), the remaining 21/27 were late device‐related complications.

The diagnostic work‐up resulted in 24/29 patients with complications in 27 revision procedures: 11 exchanges of the entire device in 11 patients, 14 revisions of the catheter in 13 patients (nine entire catheter, four distal segment, one swap of proximal segment) and two exchanges of the pump in two patients. In three patients a nonsurgical intervention was performed (application of blood patches in two patients and abdominal compression in one patient). In two patients, a surgical exploration revealed no obvious reason for the dysfunction of the entire system; thereafter, in one patient the device functioned normally again (thus catheter kinking suspected as most likely etiology), in the other patient the ITB‐pump was explanted after an interdisciplinary decision due to functional worsening of a hemispastic syndrome. In patients with a complication, the median duration of the hospital stay from admission to the consecutive interventional procedure to resolve the complication was 5 (IQR 2‐13.75) days. Further details of patients with a complication are given in Table 2.

**Table 2:** Clinical data and information on investigated risk factors of the 29 patients with 32 complications

No	Pathology	Complication	Time to event^b^ [days]	Imaging	Procedure	Median modified Rankin Scale	Median barthel index	Median flow rate [μl/hr]	Median ashworth scale	Catheter
1	SP	Catheter dislocation of distal segment	64	XR: dislocation	Exchange of entire catheter	2	95	9.2	4	8731SC
2	TBI	Catheter dislocation of distal segment	914	CT: dislocation	Exchange of entire catheter	5	0	5.8	4	8731SC
3	TSI	CSF leakage (early)	24	No imaging	Patching	3	80	25.4	4	8731SC
4	Hypoxia	CSF leakage (late)	1,822	No imaging	Exchange of entire system	4	60	18.6	4	8709/8596
5^a^	Disc herniation	Catheter disconnection	737^d^	No imaging	Exchange of entire catheter	4	65	10.8	0	8709/8596
6^a^	Disc herniation	Pump site infection	884^d^	No imaging	Exchange of pump	4	65	5.1	0	8709/8596
7	ICH	Pump site infection	168	No imaging	Explantation of pump	4	45	6.7	4	8731SC
8	TBI	Catheter kinking	19^c^	XR+CA: no signs	No revision	4	0	4.6	4	8731SC
9	Spinal ischemia	Unknown catheter dysfunction	2,471	Fluoroscopy: No signs	Exchange of entire system	4	30	7.1	4	8731SC
10	TBI	Catheter kinking	115	XR+CA: no signs	Exchange of distal segment	5	0	22.9	5	8711
11^a^	TSI	Catheter dislocation of distal segment	32	No imaging	Exchange of entire catheter	4	75	19.2	3	8731SC
12^a^	TSI	Catheter dislocation of distal segment	28^d^	XR: no signs	Exchange of entire catheter	4	75	19.2	3	8731SC
13	MS	Catheter kinking	364	XR & CT+CA: no signs	Exchange of entire catheter	5	20	2.7	4	8781
14	TSI	Catheter dislocation of distal segment	657	XR: catheter at L3 CT+CA: no signs	Exchange of distal segment	4	75	2.7	1	8731SC
15	CP	Pump site infection	26	No imaging	Exchange of entire system	5	0	4.2	4	8781
16	Hypoxia	Pump site infection	368	No imaging	Exchange of entire system	5	0	8.3	5	8731SC
17	SAH	Catheter dislocation of distal segment	25	CT+CA: dislocation Fluoroscopy: dislocation	Exchange of distal segment	5	0	3.1	4	8711
18	ICH	Catheter disconnection	2,470^d^	XR: disconnection	Exchange of pump and proximal segment	4	40	2.3	4	8709/8596
19	TBI	Catheter dislocation of distal segment	838^d^	Fluoroscopy: no signs	Exchange of entire catheter	5	0	2.3	5	8711
20	SP	Unknown catheter dysfunction	1,174	MRI: no signs	Exchange of distal segment	1	100	22.5	5	8781
21	Hypoxia	Pump site infection	23	No imaging	Exchange of entire system	5	0	39.2	5	8781
22	MS	Catheter disconnection of proximal segment	33	CT: no signs	Exchange of proximal segment	5	5	7.5	5	8781
23	Hypoxia	CSF leakage (early)	16	No imaging	Extracorporal compression	5	0	6.3	4	8731SC
24	TSI	Catheter disconnection	1,864^d^	XR & CT: no signs	Exchange of entire catheter	4	75	11.9	0	8711
25	SP	Catheter break	1,570^d^	CT+CA: no intradural contrast	Exchange of entire system	2	85	14.6	4	8731SC
26	SP	CSF leakage (early)	44	MRI: leakage	Patching	1	100	2.7	3	8781
27	TBI	Catheter dislocation of distal segment	2,058	CT+CA: no signs	Exchange of entire system	5	0	7.2	5	8731SC
28	SP	Catheter disconnection	2,179^d^	XR: disconnection CT: loop	Exchange of entire system	2	100	11.0	3	8731SC
29^a^	TSI	CSF leakage (late)	2,200^d^	XR + CA: no signs CT + CA: leakage	Exchange of entire system	4	60	5.2	0	8731SC
30^a^	TSI	Pump hypermobility	1,683^c^	No imaging	Pump exchange	4	67.5	10	0	8731SC
31	TSI	Catheter dislocation of distal segment	12^d^	No imaging	Exchange of entire catheter	4	80	11.2	0	8731SC
32	SAH	CSF leakage (late)	2,270	XR: no signs	Exchange of pump and proximal segment	4	10	8.3	3	8731SC

CA, contrasting agent; CP, cerebral palsy; CSF, cerebrospinal fluid; CT, computer tomography; MRI, magnet resonance imaging; MS, multiple sclerosis; No, number of complication; SAH, subarachnoidal hemorrhage; SP, spastic paralysis; TBI, traumatic brain injury; TSI, traumatic spinal injury; XR, conventional x‐ray.

a 2 complications in 3 patients each.

b Time to event was calculated from the last surgical procedure.

c First implantation or revision surgery.

d Scheduled device swap due to end of battery life to the date of a consecutive intervention (surgical or non‐surgical interventions).

**Figure 1:**
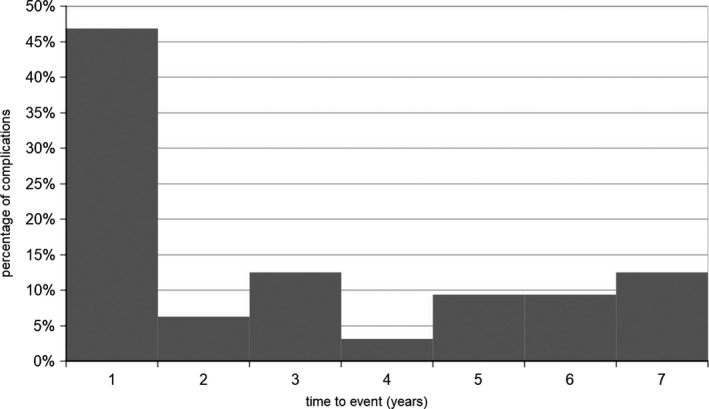
Temporal distribution of occurring complications within the first 7 years after the last intrathecal baclofen (ITB)‐related surgical procedure (first implantation surgery, replacement surgery due to end of battery life or due to a revision after a complication)

**Figure 2:**
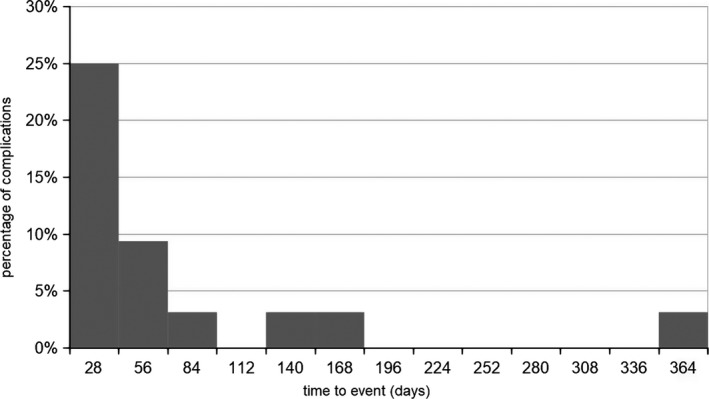
Temporal distribution of occurring complications within the first 12 months after the last surgical procedure (first implantation surgery, replacement surgery due to end of battery life or due to a revision after a complication)

The complication rate after implantation of an ITB‐pump was 0.063 per pump year for any type of complication in the 11‐year follow‐up of our patient group, for surgical‐related complications 0.01 per pump year and for device‐related problems 0.053 per pump year respectively. Per implantation, the complication rate was 0.24. Twenty of 32 complications occurred after the first implantation of the device, and 12/32 complications after scheduled replacement surgeries due to end of battery life or replacement surgeries due to revision. Here, statistical analysis revealed no significant increase in complications after the initial implantation of the device compared to replacement surgeries. The median time from the last ITB‐related surgery to the first complication was 18 (IQR 1‐57) months. Overall, 15/32 (47%) complications occurred within the first 12 months after the last surgical intervention regarding the ITB system, thereof 8/32 (25%) complications in the first month (5 after first implantation, 3 after replacement) (Figures 1 and 2).

### 3.2 Risk factors for complications

Analysis revealed a significant association between occurrence of a complication and a spinal localization of lesion responsible for the spasticity (OR 2.71, *p* = .021), whereas sex (*p* = .663, age (*p* = .304) were not significantly related to a complication. Furthermore, there was a significant difference regarding the independence in activities of daily life in patients with complications compared to patients without complications (Barthel Index 53 [IQR 0‐75] vs. 0 [IQR 35‐0]; OR 2.84, *p* = .006). Patients with a complication also differed significantly in the modified Rankin Scale from patients without a complication (4 [IQR 4‐5] vs. 5 [IQR 4‐5]; OR 2.86, *p* = .015). No significant differences in both patient groups were found regarding spasticity (Ashworth Scale 4 vs. 3, *p* = .381) and flow rate (5.6 μl/hr vs. 7.9, *p* = .065).

A subgroup analysis on the 21 patients with 22 catheter‐related complications revealed that other catheter types than an Ascenda^®^ catheter (introduced in 2010, Model 8781) were also identified as a risk factor (19/73, 26% vs. 3/36, 8%; OR 3.87, *p* = .041).

## 4 DISCUSSION

This study provides a systematic assessment of procedure‐ and device‐related complications after implantation of an ITB‐pump in a large patient cohort. Twenty‐five per cent of the patients had complications, most frequently catheter‐associated problems in our series, followed by procedure‐ and pump‐related complications. Risk factors were a spinal lesion, older catheter types, a lower modified Rankin Scale and a higher Barthel Index. About half of the complications occurred within the first 12 months after any ITB‐related surgery, half of these occurring within the first month.

Comparison of our results with previously published papers is limited due to the variety of different methodologies, study‐populations, included types of adverse events and duration of follow‐up. The review of 32 full‐length manuscripts and 10 case reports published by Stetkarova and co‐workers in 2010 also focused exclusively on procedure‐, pump‐, and catheter‐related complications of ITB‐administration (Stetkarova et al., 2010). Complication rates varied widely among the studies with a mean complication rate of 0.41 per implantation but ranging from 0‐2.24 per implantation across the reviewed studies. The complication rate after implantation of an ITB‐pump was 0.24 per implantation in our study cohort, thus lying within the reported range. The authors found higher complication rates in centers with a longer mean follow‐up than 18 months (0.56 ± 0.56 vs. 0.23 ± 0.19). With regard to our mean follow‐up of 56 months, our complication rate lies at the lower part of the range.

Based on the results of our study, we found a nearly threefold increased risk of a complication in patients with a spinal localization of lesion responsible for the spasticity versus supraspinal localization. This is in contrast with Borrini and coworkers who reported no significant correlation between localization of lesion and occurrence of adverse events (Borrini et al., 2014). The reason for this discordant finding is not clear, possibly methodological differences might play a role as the study period was only 1 year and median follow‐up duration was substantially shorter (10 months vs. 42 months in our cohort). In our study group, a higher Barthel Index regarding the independence in activities of daily life and a lower modified Rankin Scale related to the ambulatory status were both associated with a nearly threefold increased risk for sustaining a complication. This is also in contrast with Borrini and coworkers who did not find a significant correlation with the ambulatory status. The results of our study might be supported by findings of a Russian working group analysing complications in 12/52 (23%) patients after ITB‐device implantation (Paskhin, Dekopov, Tomsky, Isagulyan, & Salova, 2017): 5/12 complications were caused by catheter migration and the authors identified patients with severe dystonia of the trunk muscles to have an increased risk of spinal catheter migrations. Accordingly, the degree of independence and mobility represented with the Barthel Index and the modified Rankin Scale might be correlated with the occurrence of catheter problems based on mechanical stress.

In our cohort, no significant association was found between occurrence of an adverse event and gender, which is in line with a previous report (Borrini et al., 2014). Also age was no risk factor for a complication in our study which is in contrast with a previously published paper (Motta et al., 2007): in the study of Motta and coworkers, complications were statistically more likely in patients with an age of 10 years and younger. This might be related to the by far lower mean age of 13.7 ± 5.7 years of the 200 children and adolescents in their study compared to ours (mean age 39 ± 16.7 years). An Ashworth Scale higher than 3 was a further risk factor in this study whereas we did not find a significant difference of the Ashworth Scale in patients with and without complications. The divergent results might be explained by the different patient population, as about 90% of included patients in the study of Motta and coworkers were affected by cerebral palsy with thus clinically dominating dystonia, whereas we included a more mixed patient population with a traumatic etiology in more than half of the patients.

The impact of dose and concentration of intrathecally administered drugs on catheter tip granuloma is still discussed controversially. A direct correlation with high concentrations or high dose opiate drugs was suggested (Coffey & Burchiel, 2002; Yaksh et al., 2002), but also an association between intrathecal granulomas and low doses and concentrations of hydromorphone (Veizi et al., 2016) reported. We analyzed a possible association between the flow rate of ITB and possibly mechanically driven complications and found no relation to occurring complications.

Motta and coworkers compared complications before and after the introduction of the Ascenda^®^ catheter for the first time in paediatric patients recently (Motta & Antonello, 2016): 120/416 (29%) children with silicone catheters sustained major complications (infections, leakages, catheter‐related problems), but only 1/92 children with an Ascenda^®^ catheter (leakage). We could confirm these results in our study as an implanted Ascenda^®^ catheter was associated with an approximately fourfold decreased risk for catheter‐related complications, most likely the consequence of technological improvements of the Ascenda^®^ catheter.

In our cohort, half of the complications occurred within the first 12 months after any ITB‐related surgery, after primary implantation procedures as well as replacement procedures due to end of battery life or revision likewise, within the first month a quarter of the complications. Also Motta and coworkers did not observe a different event rate after initial device‐implantations compared to complications after replacement surgeries (Motta & Antonello, 2014). Thus, the critical period is the first year and especially the first month after any ITB‐related surgery.

### 4.1 Study limitations

Limitations of our study are: First of all, the retrospective assessment of the data has to be taken into account. Furthermore, the investigated patients of our rehabilitation center during the study period comprise a mixed patient population regarding the different etiologies of spasticity. Moreover, the follow‐up periods varied widely among the patients as all patients with an intrathecal ITB‐device and any treatment at our department (once only admissions as well as regular admissions) during the observational period were included.

Care of patients with an ITB‐device requires a highly specialized, interdisciplinary operating, experienced staff of neurologists, neurosurgeons, nurses, and physiotherapists. Knowledge of adverse events and their risk factors is necessary for patient counseling about risks and benefits before and adequate care after the implantation of the ITB‐device. A spinal localization of lesion, other catheter types than an Ascenda^®^ catheter, a lower modified Rankin Scale and a higher Barthel Index are most predictive for sustaining a complication. Based on the results of our study, the following measures might be taken to avoid as well as identify complications as early as possible: 1. Appropriate care of high‐risk patients i.e. (i) patients with a spinal localization of lesion combined with a higher Bartel Index, possibly related to the ability to participate in the extended activities of daily life and (ii) patients with less impairment of the ambulatory status and therefore, a higher grade of trunk mobility 2. Use of newer catheter types which might be helpful to reduce complications according to improvements of the catheter material. 3. The temporal distribution of complications might be helpful when scheduling postsurgical care and follow‐up after the first implantation of the ITB‐device as well as any replacement surgery.

Further prospective multicenter studies are necessary to evaluate risks and safety issues of treatment with an ITB‐device in order to improve the risk management of complications related to the ITB‐device.

## CONFLICT OF INTEREST

None declared.
